# Evaluating the relationship between clinical and demographic characteristics of insulin-using people with diabetes and their health outcomes: a cluster analysis application

**DOI:** 10.1186/s12913-021-06603-0

**Published:** 2021-07-08

**Authors:** Elizabeth L. Eby, Alison Edwards, Eric Meadows, Ilya Lipkovich, Brian D. Benneyworth, Kenneth Snow

**Affiliations:** 1 Eli Lilly and Company, Lilly Corporate Center, Indianapolis, IN 46285 USA; 2 Healthagen LLC (renamed CVS Health Clinical Trial Services LLC, effective 01 November 2020), 151 Farmington Avenue, Hartford, CT 06156 USA

**Keywords:** Healthcare claims data, Healthcare utilization, Subgroup identification, Diabetes management

## Abstract

**Background:**

The aim of this study was to determine how clusters or subgroups of insulin-treated people with diabetes, based upon healthcare resource utilization, select social demographic and clinical characteristics, and diabetes management parameters, are related to health outcomes including acute care visits and hospital admissions.

**Methods:**

This was a non-experimental, retrospective cluster analysis. We utilized Aetna administrative claims data to identify insulin-using people with diabetes with service dates from 01 January 2015 to 30 June 2018. The study included adults over the age of 18 years who had a diagnosis of type 1 (T1DM) or type 2 diabetes mellitus (T2DM) on insulin therapy and had Aetna medical and pharmacy coverage for at least 18 months (6 months prior and 12 months after their index date, defined as either their first insulin prescription fill date or their earliest date allowing for 6 months’ prior coverage). We used K-means clustering methods to identify relevant subgroups of people with diabetes based on 13 primary outcome variables.

**Results:**

A total of 100,650 insulin-using people with diabetes were identified in the Aetna administrative claims database and met study criteria, including 11,826 (11.7%) with T1DM and 88,824 (88.3%) with T2DM. Of these 79,053 (78.5%) people were existing insulin users. Seven distinct clusters were identified with different characteristics and potential risks of diabetes complications. Overall, clusters were significantly associated with differences in healthcare utilization (emergency room visits, inpatient admissions, and total inpatient days) after multivariable adjustment.

**Conclusions:**

This analysis of healthcare claims data using clustering methodologies identified meaningful subgroups of patients with diabetes using insulin. The subgroups differed in comorbidity burden, healthcare utilization, and demographic factors which could be used to identify higher risk patients and/or guide the management and treatment of diabetes.

**Supplementary Information:**

The online version contains supplementary material available at 10.1186/s12913-021-06603-0.

## Introduction/background

Diabetes is a complex, chronic illness that affects about 30 million people or 9.4% of the population in the United States [1, 2]. The population with diabetes continues to grow and the percentage of adults with diabetes increases with age, where approximately 25% of all adults over 65 years of age have diabetes [3]. Diabetes remains the seventh leading cause of death in the United States with approximately 80,000 death certificates in 2015 listing diabetes as the underlying cause of death [3], although the actual number of diabetes-associated deaths may be higher as diabetes is often underreported as a cause of death [3].

The majority of people with diabetes are classified as having type 2 diabetes mellitus (T2DM), occurring in about 90 to 95% of diagnosed cases [4, 5], with approximately 5% of diagnosed cases classified as type 1 diabetes mellitus (T1DM) [4]. T1DM is characterized by the lack of insulin production, and as such patients require insulin to survive [5]. T2DM is characterized by the body’s resistance to insulin action in addition to a relative insulinopenia [6], and is usually diagnosed in adults. Genetic factors and lifestyle play an important role in disease progression for people with either T1DM or T2DM [4].

People with diabetes visit physician offices and emergency rooms (ER) more frequently than people without diabetes, are more likely to be admitted to the hospital, and more commonly receive home health care [7]. The financial burden of diabetes to individuals and society is estimated to be a total of $327 billion, including $237 billion in direct costs and $90 billion in reduced productivity in 2018 [3, 7]. Healthcare expenditures are about 2.3 times higher for people with diabetes than people without diabetes with an average medical diabetes-related expenditures of approximately $9600 per year in the United States [7].

Although all people with T1DM use insulin, insulin use varies among people with T2DM. Most are initially managed with oral hypoglycemic agents but over time, most will ultimately utilize insulin. The majority of the diabetic population uses injection-based administration with insulin pens or vials and syringes while the remainder uses insulin pumps (continuous subcutaneous insulin infusion) [8, 9]. Since 2005, there has been a significant increase in the use of insulin pens while the use of vials and syringes to deliver insulin has decreased over time [10]. Insulin pens have simplified the administration of insulin, resulting in more accurate and easier delivery of insulin relative to vials and syringes [11]. Pumps deliver rapid-acting insulin throughout the day. Diabetes-related technologies are available to monitor blood glucose and include the standard blood glucose monitor (single reading) and continuous blood glucose monitoring systems (real time and intermittently scanned) [8].

In 2005–2012, among patients who had any insulin use, only 31.4% had an hemoglobin A1c (HbA1c) < 7%; therefore, as glycemic control is still not attained by most people with diabetes, there is a need for new approaches to identify subgroups of people with diabetes (T1DM or T2DM) who might have risk factors that could impact treatment decisions and targeted disease management [12]. Given the differences across the US population especially in disease severity and utilization of healthcare, we sought insight from a large, real-world database utilizing Aetna’s administrative claims. In this study, we used clustering techniques to identify subgroups of people treated with insulin based upon healthcare resource utilization, select social demographic and clinical characteristics, and diabetes management parameters. We then related these subgroups to health outcomes, including ER visits, hospital admissions, and total inpatient days.

## Methods

### Study design and data sources

We used a non-experimental, retrospective design in this study utilizing Aetna’s administrative claims data containing membership, eligibility, medical claims, pharmacy claims, laboratory test results, and data derived for Aetna’s care management processes (Health Profile Database [13]) for Aetna fully insured Commercial and Medicare Advantage members, with services dates from 01 January 2015 to 30 June 2018. All of the data used in this study were fully de-identified and Health Insurance Portability and Accountability Act compliant.

### Sample selection and patient population

**Fig. 1:**
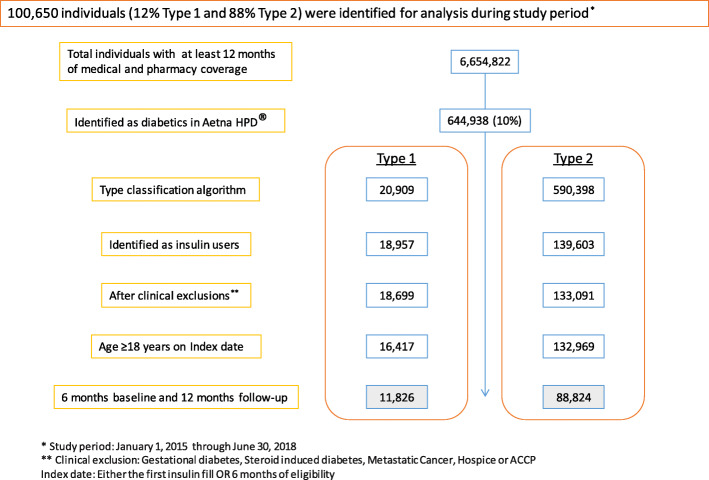
Member selection - population funnel

Using the International Classification of Diseases Ninth and Tenth Revision (ICD9-CM v1 and ICD10-CM) diagnostic codes, the study included adults 18 years of age and older who had a diagnosis of T1DM or T2DM who utilized insulin therapy and had Aetna medical and pharmacy coverage for at least 18 continuous months (6 months of coverage prior and 12 months after their index date, defined as either their first insulin prescription fill date or their earliest insulin fill date allowing for 6 months prior coverage; Fig. 1). People with diabetes were excluded from the analyses if any of the following criteria were met during the entire study period: had ≥1 inpatient or outpatient medical claim with a diagnosis in any position of gestational diabetes, steroid-induced diabetes, or metastatic cancer; had indications of hospice use; or were enrolled in Aetna’s Compassionate Care Program. The study was approved by the Sterling Institutional Review Board (Atlanta, Georgia, USA).

### Statistical analysis

Descriptive analyses were performed initially on the overall study population and for T1DM and T2DM separately. After clustering, descriptive analyses were completed for each cluster. Descriptive statistics were generated for all demographic characteristics and utilization measures as applicable to the type of variable. Continuous variables were described using means with standard deviations (SD) or medians with first and third quartiles if data were highly skewed. Categorical and binary variables were described using counts and percentages. Healthcare utilization were reported as means and SD and number of patients with ≥1 visit.

### Cluster analysis

A cluster analysis was performed on 13 pre-period variables that were hypothesized to be both related to outcomes and to the use of diabetes technology (blood glucose monitor [BGM], continuous glucose monitoring [CGM], or insulin pumps). Variables were identified by the study team based on clinical opinion and were limited to variables found in the administrative claims database. They included age, number of endocrinologist visits, diabetes complications severity index (DCSI) [14], Charlson comorbidity index (CCI) [15], number of HbA1c tests, number of months on insulin, number of ER visits, total number of inpatient days, number of discordant comorbidities, number of concordant comorbidities, number of medical claims, proportion diabetes-related claims, and estimated income. Concordant comorbidities were defined as those that share the same pathophysiologic risk profile and management plan as with diabetes, whereas discordant conditions are those that are not directly related to diabetes in either their pathophysiology or management [16]. As the underlying pathophysiology differs for T1DM and T2DM, concordant and discordant were defined separately for each type of diabetes (see Supplemental Materials Table S1). Clinically dominant comorbidities, those that are so complex or serious that they tend to eclipse the management of other conditions [16] such as end stage renal failure or dementia, were considered but not found in the study population. Claims were considered diabetes related if they contained a diagnosis for diabetes in any position. All 13 cluster variables (mentioned above) had significant bivariate associations with utilization of diabetes technology and were standardized according to the type of variable. We opted to use variables associated with the utilization of diabetes technology in the cluster analysis to identify unexpected patterns in the characteristics of patients who utilized different types of technology. After clustering, utilization of diabetes technology along with other relevant variables such as diabetes type, insurer, and HbA1c levels (when available, for 31.2% of the population) were reported to help characterize the clusters.

We utilized K-means methodology to identify the clusters based on the following 13 variables: age, number of endocrinologist visits, DCSI, CCI, number of HbA1c tests, number of months on insulin, number of ER visits, total number of inpatient days, number of discordant comorbidities, number of concordant comorbidities, number of medical claims, proportion diabetes-related claims, and estimated income. To determine the optimal number of clusters, we used the Jump method [17], a non-parametric method for choosing the number of clusters based on “distortion” which is a measure of within-cluster dispersion. We chose the Jump method as it is a simple and readily available method that has performed well against other competing methods in simulated data analyses [17].

To identify which variables played a key role in the formation of clusters and to obtain simple descriptive rules for clusters, classification and regression trees (CART) were used with cluster assignment as the “outcome” variable and all variables used in clustering as the “predictors.” This analysis was performed using the Ctree function in the R package partykit.

Outcomes of interest, measured in the 12-month follow-up period, included: all-cause ER utilization, all-cause inpatient hospitalization, and total inpatient days. To determine if the clusters were associated with the outcomes measured during the study, multivariable generalized linear models were applied with clusters as the independent variables and other covariates or interactions to account for confounding. The generalized linear models included binomial distribution and logit link function (ER and inpatient hospitalization outcomes) and negative binomial distribution with log link function (total inpatient days outcome). For the multivariable regression analyses, we used a stepwise selection methodology (or backward elimination) with variable removal when *p* ≥ 0.05. Akaike Information Criterion was also used to determine the best fit model. All statistical analyses were conducted using SAS Enterprise Guide Version 6.1 (SAS Institute Inc., Cary, NC, USA) or R version 3.2.5 (R Foundation for Statistical Computing, Vienna, Austria).

## Results

### Study population

A total of 100,650 insulin-using people with diabetes met the study criteria, including 11,826 (11.7%) with T1DM and 88,824 (88.3%) with T2DM. Table 1 shows the pre-period demographic characteristics for the total population and differences between people with T1DM and T2DM. The majority of study patients (78.5%) were existing insulin users at the index date, whereas the remaining 21.5% were new insulin initiators. The mean age (SD) was 62.2 (14.2) years with a mean age (SD) of 45.3 (16.3) years for those with T1DM and 64.4 (12.3) years for those with T2DM. There were slightly more males than females (51.6% male versus 48.3% female). The median (interquartile range) household income was $54,143 ($43,184 to $69,606) for the total population with a slightly higher income for those with T1DM of $63,304 ($49,195 to $81,547) compared to T2DM of $53,299 ($42,675 to $68,059).

**Table 1:** Pre-period^1^ demographic and clinical characteristics (baseline)

	Total		T1DM		T2DM		p
	N	%	N	%	N	%	
**Total Number of People With Diabetes**	100,650	100%	11,826	11.7%	88,824	88.3%	–
**Subgroup of Existing Insulin Users During the Pre-period**	79,053	78.5%	10,975	92.8%	68,078	76.6%	< 0.0001
**Characteristics**							
**Age (n, %)**							< 0.0001
18–40	8068	8.0%	4843	41.0%	3225	3.6%	
41–60	32,960	32.7%	4713	39.9%	28,247	31.8%	
> 60	59,622	59.2%	2270	19.2%	57,352	64.6%	
**Age (mean, SD)**	62.18	14.24	45.26	16.27	64.43	12.30	< 0.0001
**Gender (n, %)**							0.0003
Male	51,983	51.6%	6290	53.2%	45,693	51.4%	
Female	48,651	48.3%	5532	46.8%	43,119	48.5%	
Missing	16	0.0%	4	0.0%	12	0.0%	
**Business Line (n, %)**							< 0.0001
Commercial	43,573	43.3%	9566	80.9%	34,007	38.3%	
Medicare advantage	57,077	56.7%	2260	19.1%	54,817	61.7%	
**Household Income, in US Dollars**							
**n (%)**	100,196	99.5%	11,801	99.8%	88,395	99.5%	
Median, Q1 - Q3	$54,143	$43,184 - $69,606	$63,304	$49,195 - $81,547	$53,299	$42,675 - $68,059	< 0.0001
**Medical Claims**							
**Number of Medical Claims (median, Q1 - Q3)**	7.00	4.00–15.00	5.00	3.00–10.00	8.00	4.00–15.00	< 0.0001
**Proportion of Medical Claims** ^**2,3**^ **That are Diabetes Related (mean, SD)**	0.54	0.31	0.60	0.33	0.52	0.30	< 0.0001
**Number of HbA1c Tests**							
**One or More Tests Performed (n, %)**	60,668	60.3%	7137	60.4%	53,531	60.3%	0.86
Mean, SD	0.91	0.93	0.89	0.88	0.91	0.94	0.0007
**Latest HbA1c Value**							
**n (%)**	31,410	31.2%	3417	28.9%	27,993	31.5%	
Mean, SD	8.84	1.94	8.38	1.64	8.89	1.96	< 0.0001
**Insulin Administration and Diabetes Technology (n, %)**							< 0.0001
CGM with pump	1974	2.0%	1734	14.7%	240	0.3%	
CGM with pen	800	0.8%	342	2.9%	458	0.5%	
CGM with vial	277	0.3%	200	1.7%	77	0.1%	
BGM with pump	2098	2.1%	1516	12.8%	582	0.7%	
BGM with pen	23,078	22.9%	2032	17.2%	21,046	23.7%	
BGM with vial	8380	8.3%	1078	9.1%	7302	8.2%	
No CGM or BGM/pump	2410	2.4%	1236	10.5%	1174	1.3%	
No CGM or BGM/pen	44,108	43.8%	2208	18.7%	41,900	47.2%	
No CGM or BGM/vial	17,525	17.4%	1480	12.5%	16,045	18.1%	
**Type of Insulin (n, %)**							< 0.0001
N	77,912	77.4%	10,524	89.0%	67,388	75.9%	
Basal only	31,917	41.0%	893	8.5%	31,024	46.0%	
Bolus only	8949	11.5%	4742	45.1%	4207	6.2%	
Both	37,046	47.5%	4889	46.5%	32,157	47.7%	
**Months on Insulin (mean, SD)**	3.99	3.48	4.81	3.26	3.89	3.49	< 0.0001
**Clinical Characteristics**							
**Number of Concordant Comorbidities (mean, SD)**	4.20	2.57	0.87	1.17	4.64	2.38	< 0.0001
**Number of Discordant Comorbidities (mean, SD)**	1.07	1.11	1.83	1.62	0.97	0.98	< 0.0001
**Charlson Comorbidity Index (CCI) (mean, SD)**	1.86	1.60	1.40	1.21	1.92	1.63	< 0.0001
**Diabetes Complication Severity Index (DCSI) (mean, SD)**	1.07	1.47	0.58	1.09	1.13	1.50	< 0.0001

Abbreviations: BGM = blood glucose monitoring; CGM = continuous glucose monitoring; HbA1c = hemoglobin A1c; n, N = number of people with diabetes; Q1 = first quartile; Q3 = third quartile; SD = standard deviation; T1DM = type 1 diabetes mellitus; T2DM = type 2 diabetes mellitus; US = United States

^1^ Pre-period was defined as 6 months prior to index date

^2^ Proportion is set to zero for those members who had zero medical claims overall during the baseline period

^3^ Diabetes–related determined by diagnosis in any diagnostic position on a medical claim

Overall, the mean (SD) CCI score was 1.9 (1.6) and the mean DCSI was 1.1 (1.5), both of which were lower for those with T1DM (Table 1). A total of 60,668 people (60.3%) had one or more HbA1c test performed and the average HbA1c value was 8.8% among those individuals (31.2%) with values in the database. Approximately 6.4% of the population used an insulin pump (37.9% T1DM and 2.2% T2DM) and 67.5% used a pen (38.7% T1DM and 71.4% T2DM). Blood glucose meters were utilized by approximately one-third of the population (33.3%). On the other hand, CGMs were only used by 3.0% overall, where T1DM patients had greater use compared to those with T2DM (19.2 and < 1%, respectively).

The healthcare utilization over the 6-month pre-period is shown in Table 2. The majority of the population had primary care physician (PCP) visits (72.3%), but the proportion with endocrinologist and cardiologist visits was lower at 21.3 and 17.8%, respectively. Inpatient hospitalizations occurred for 14.0% of the population and 18.8% had ER visits. Overall, people with T1DM had lower utilization of all-cause and diabetes-related healthcare services than people with T2DM, with the exception of endocrinologist visits.

**Table 2:** Pre-period^1^ healthcare utilization (baseline)

–.	Total	T1DM		T2DM		p	
–	N	%	N	%	N	%	
**Total Number of People With Diabetes**	**100,650**	**100%**	**11,826**	**11.7%**	**88,824**	**88.3%**	**–**
**Healthcare Resource Utilization**^**2**^							
**All-cause Utilization**							
All-cause ER visits (*n* > 0, %)	18,930	18.8%	1452	12.3%	17,478	19.7%	< 0.0001
All-cause ER visits (mean, SD)	0.30	0.85	0.18	0.59	0.32	0.88	< 0.0001
All-cause IP visits (n > 0, %)	14,048	14.0%	761	6.4%	13,287	15.0%	< 0.0001
All-cause IP visits (mean, SD)	0.21	0.64	0.09	0.40	0.23	0.67	< 0.0001
All-cause IP days (mean, SD)	1.74	8.24	0.44	3.45	1.92	8.67	< 0.0001
All-cause IP days for patients with ≥1 admit (mean, SD)	12.48	18.79	6.79	11.94	12.80	19.05	< 0.0001
All-cause PCP visits (n > 0, %)	72,779	72.3%	6569	55.5%	66,210	74.5%	< 0.0001
All-cause PCP visits (mean, SD)	2.05	2.27	1.20	1.66	2.16	2.32	< 0.0001
All-cause Endocrinologist visits (n > 0, %)	21,436	21.3%	5055	42.7%	16,381	18.4%	< 0.0001
All-cause Endocrinologist visits (mean, SD)	0.36	0.80	0.69	0.97	0.31	0.76	< 0.0001
All-cause Cardiologist visits (n > 0, %)	17,954	17.8%	884	7.5%	17,070	19.2%	< 0.0001
All-cause Cardiologist visits (mean, SD)	0.30	0.83	0.11	0.50	0.32	0.87	< 0.0001
No visit to PCP, Endocrinologist, or Cardiologist (n, %)	18,671	18.6%	2677	22.6%	15,994	18.0%	< 0.0001
**Diabetes-related Utilization**^**3**^							
Diabetes-related ER visits (n > 0, %)	15,057	15.0%	1163	9.8%	13,894	15.6%	< 0.0001
Diabetes-related ER visits (mean, SD)	0.23	0.71	0.14	0.51	0.24	0.73	< 0.0001
Diabetes-related IP visits (n > 0, %)	13,536	13.4%	750	6.3%	12,786	14.4%	< 0.0001
Diabetes-related IP visits (mean, SD)	0.20	0.60	0.08	0.39	0.21	0.62	< 0.0001
Diabetes-related IP days (mean, SD)	1.59	7.58	0.40	3.15	1.74	7.98	< 0.0001
Diabetes-related IP days for patients with ≥1 admit (mean, SD)	11.79	17.53	6.37	10.90	12.11	17.79	< 0.0001

Abbreviations: ER = emergency room; HbA1c = hemoglobin A1c; IP = inpatient; n, N = number of people with diabetes; PCP = primary care physician; Q1 = first quartile; Q3 = third quartile; SD = standard deviation; T1DM = type 1 diabetes mellitus; T2DM = type 2 diabetes mellitus

^1^ Pre-period was defined as 6 months prior to index date

^2^ Statistical comparisons for average utilization were conducted using Wilcoxon Rank Sum tests

^3^ Diabetes–related determined by diagnosis in any diagnostic position on a medical claim

### Cluster formation and analysis

**Fig. 2:**
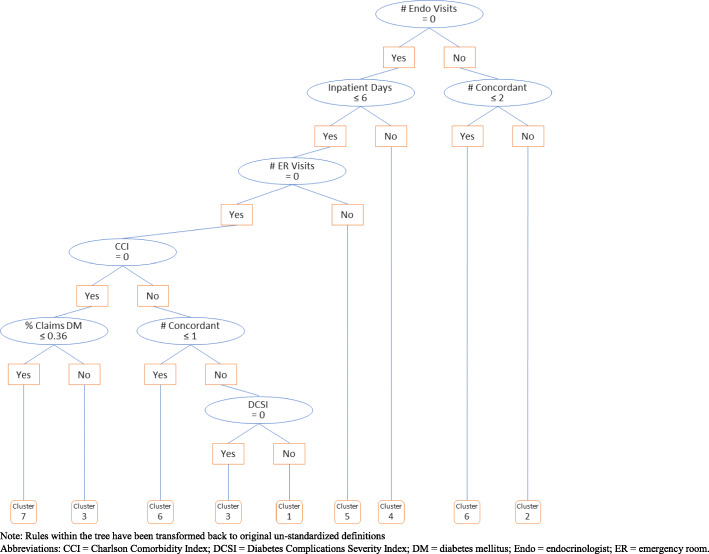
Cluster analysis schematic. Note: Rules within the tree have been transformed back to original un-standardized definitionsAbbreviations: CCI = Charlson Comorbidity Index; DCSI = Diabetes Complications Severity Index; DM = diabetes mellitus; Endo = endocrinologist; ER = emergency room

We identified seven clusters of people with diabetes, which had distinguishable characteristics and risk factors. Characteristics of the seven identified clusters are shown in Table 3. Seven of the 13 clustering variables studied using the CART analysis were identified as being the most important factors for cluster formation. These included number of endocrinology visits, total inpatient days, concordant comorbidities, number of ER visits, comorbidity burden as measured by CCI and DCSI scores, and percentage of diabetes-related medical claims. Figure 2 illustrates the CART analysis after the clusters were formed.

**Table 3:** Description of people with diabetes identified through cluster analysis

	CLUSTER NUMBER						
	1	2	3	4	5	6	7
**Cluster Label**	Low ER Utilizers with High Comorbid Burden	Endocrinology Utilizers with Concordant Comorbidities	Low ER Utilizers with High Diabetes-related Claims without Discordant Comorbidities	High Inpatient Utilizers with High Comorbid Burden	High ER Utilizers	Endocrinology Utilizers without Concordant Comorbidities	Lowest Overall Utilizers
**Number of People (n, %)**	22,508 (22.4%)	13,986 (13.9%)	22,880 (22.7%)	5629 (5.6%)	11,351 (11.3%)	10,049 (10.0%)	14,247 (14.2%)
**Age (mean, SD) (years)**	69.80 (9.85)	65.16 (10.83)	60.71 (10.83)	69.54 (11.98)	64.92 (12.54)	38.34 (12.16)	61.31 (12.51)
**Male (%)**	48.0%	50.8%	45.4%	48.9%	54.8%	43.0%	49.5%
**Household Income (US dollars)**^**1**^ **Median (Q1 - Q3)**	$51,665 ($42,344–$65,516)	$59,733 ($47,149–$77,449)	$51,879 ($42,139–$66,124)	$53,612 ($43,017–$68,301)	$49,154 ($40,163–$61,588)	$65,972 ($51,625–$84,930)	$54,107 ($43,152–$69,429)
**Medicare Advantage (%)**	80.3%	62.2%	42.6%	81.5%	72.5%	3.8%	51.7%
**Diabetes Type T1DM (%)**	3.3%	13.6%	2.2%	2.1%	3.6%	69.6%	8.1%
**Number of HbA1c Tests**							
One or more HbA1c tests performed (%)	66.4%	75.8%	71.3%	54.3%	63.8%	63.0%	15.2%
Mean (SD)	1.01 (0.91)	1.29 (1.00)	1.02 (0.83)	0.89 (1.21)	1.00 (0.99)	0.87 (0.81)	0.17 (0.41)
**Latest HbA1c Value**							
N	7007	6215	7494	1471	3260	3045	2918
Mean (SD)	8.56 (1.79)	8.60 (1.69)	9.35 (2.07)	8.25 (1.81)	8.95 (2.05)	8.82 (2.02)	8.87 (1.97)
**Utilization of Diabetes Technology**							
Pump (%)	2.8%	10.6%	2.1%	4.2%	4.3%	29.2%	1.7%
BGM (%)	33.3%	38.6%	31.8%	29.6%	35.3%	39.6%	26.2%
CGM (%)	1.0%	6.8%	0.8%	1.0%	1.2%	14.5%	0.3%
**Type of Insulin (%)**							
Basal only	45.0%	27.9%	55.3%	32.5%	40.8%	17.3%	47.4%
Bolus only	6.2%	15.6%	6.0%	10.9%	8.2%	36.7%	7.2%
Both	48.7%	56.5%	38.8%	56.6%	51.0%	46.0%	45.3%
**Months on Insulin (mean, SD)**	4.45 (3.58)	5.02 (3.72)	3.34 (3.15)	2.68 (3.18)	3.70 (3.47)	4.29 (3.25)	3.87 (3.42)
**Clinical Characteristics**							
**Number of Concordant Comorbidities (mean, SD)**	5.56 (2.17)	4.56 (2.31)	3.29 (1.61)	6.72 (2.34)	5.53 (2.34)	0.52 (0.76)	3.67 (2.19)
**Number of Discordant Comorbidities (mean, SD)**	1.40 (1.07)	1.46 (1.26)	0.50 (0.74)	1.44 (1.16)	1.31 (1.14)	0.89 (1.10)	0.88 (0.99)
**Charlson Comorbidity Index (CCI) (mean, SD)**	2.70 (1.44)	2.47 (1.50)	1.15 (0.78)	3.92 (2.03)	2.47 (1.61)	1.09 (0.74)	0.30 (0.55)
**Diabetes Complication Severity Index (DCSI) (mean, SD)**	1.79 (1.39)	1.33 (1.39)	0.26 (0.58)	3.48 (1.95)	1.66 (1.53)	0.22 (0.56)	0.15 (0.49)
**Median (Q1 - Q3) Number of Medical Claims**	11.00 (7.00–18.00)	11.00 (7.00–17.00)	4.00 (2.00–6.00)	43.00 (30.00–64.00)	14.00 (9.00–23.00)	4.00 (2.00–7.00)	3.00 (0.00–6.00)
**Proportion of Medical Claims** ^**2,3**^ **That are Diabetes Related (mean, SD)**	0.46 (0.24)	0.53 (0.24)	0.78 (0.23)	0.53 (0.25)	0.51 (0.24)	0.69 (0.30)	0.19 (0.22)
**Healthcare Resource Utilization**^**4**^							
**All-cause Utilization**							
All-cause ER visits (%)	0.0%	10.7%	6.2%	45.2%	100.0%	12.0%	6.5%
All-cause ER visits (mean, SD)	0.00 (0.00)	0.12 (0.36)	0.07 (0.26)	0.82 (1.37)	1.77 (1.50)	0.15 (0.44)	0.07 (0.28)
All-cause IP visits (%)	11.6%	7.5%	5.4%	99.1%	22.2%	6.0%	3.2%
All-cause IP visits (mean, SD)	0.13 (0.36)	0.08 (0.31)	0.06 (0.25)	2.11 (1.29)	0.26 (0.53)	0.07 (0.28)	0.03 (0.20)
All-cause IP days for patients with ≥1 admit (mean, SD)	3.84 (2.04)	3.88 (2.69)	4.07 (2.81)	25.34 (24.57)	4.13 (2.75)	3.61 (2.96)	5.03 (4.14)
All-cause PCP visits (%)	83.7%	71.4%	79.8%	81.3%	84.1%	55.8%	41.8%
All-cause PCP visits (mean, SD)	2.60 (2.32)	1.73 (1.88)	1.87 (1.58)	3.44 (3.69)	3.31 (2.97)	1.10 (1.41)	0.86 (1.39)
All-cause Endocrinologist visits (%)	0.7%	100.0%	4.0%	13.5%	12.1%	39.2%	2.1%
All-cause Endocrinologist visits (mean, SD)	0.01 (0.08)	1.83 (0.94)	0.04 (0.23)	0.22 (0.69)	0.16 (0.47)	0.58 (0.85)	0.02 (0.15)
All-cause Cardiologist visits (%)	29.0%	27.5%	5.1%	36.8%	28.8%	2.0%	6.1%
All-cause Cardiologist visits (mean, SD)	0.46 (0.98)	0.43 (0.90)	0.07 (0.33)	0.79 (1.48)	0.53 (1.13)	0.03 (0.24)	0.08 (0.37)
No visit to PCP, Endocrinologist or Cardiologist (%)	12.4%	0.0%	17.4%	12.7%	11.2%	21.4%	54.6%
**Diabetes-related Utilization**^**3**^							
Diabetes-related ER visits (%)	0.0%	8.5%	5.2%	35.6%	80.8%	9.3%	4.0%
Diabetes-related IP visits (%)	11.2%	7.3%	5.4%	95.7%	21.3%	5.8%	2.7%
Diabetes-related IP days for patients with ≥1 admit (mean, SD)	3.81 (2.02)	3.87 (2.67)	4.05 (2.79)	23.64 (22.99)	4.07 (2.71)	3.52 (2.84)	4.88 (4.07)
**Cluster Characteristics**	• No patients with all-cause or diabetes-related ER visits • Second highest mean CCI and DCSI scores	• 100% had an Endocrinologist visit • Highest proportion with 1+ HbA1c tests performed (76%)	• Highest average HbA1c lab value (mean = 9.35) • Highest proportion of medical claims that were diabetes-related (mean = 0.78)	• Highest proportion of new insulin users (mean = 2.68 months) • Highest mean CCI and DCSI scores • Highest average number of medical claims (median = 43) • Nearly 100% had an Inpatient Hospitalization	• 100% had an ER visit • 81% had a diabetes-related ER visit • Higher observed HbA1c (mean = 8.95)	• Highest proportion of type 1 diabetics (70%) • Youngest (mean age = 38 years) • Relatively high proportion with an Endocrinology visit (39%) • Highest diabetes technology use (insulin pump, CGM, and/or BGM)	• Healthiest, according to CCI and DCSI scores • Largest proportion without PCP, Endocrinologist, or Cardiologist visits (55%) • Lowest total number of medical claims (median = 3)

Abbreviations: BGM = blood glucose monitor; CCI = Charlson Comorbidity Index; CGM = continuous glucose monitor; DCSI = diabetes complications severity index; ER = emergency room; HbA1c = hemoglobin A1c; IP = inpatient; n, N = number of people with diabetes; PCP = primary care physician; Q1 = first quartile; Q3 = third quartile; SD = standard deviation; T1DM = type 1 diabetes mellitus

^1^ Missing values for median household income were imputed for 454 patients for the purpose of cluster analysis

^2^ Proportion is set to zero for those members who had zero medical claims overall during the baseline period

^3^ Diabetes–related determined by diagnosis in any diagnostic position on a medical claim

^4^ Statistical comparisons for average utilization were conducted using Kruskal-Wallis tests

**Fig. 3:**
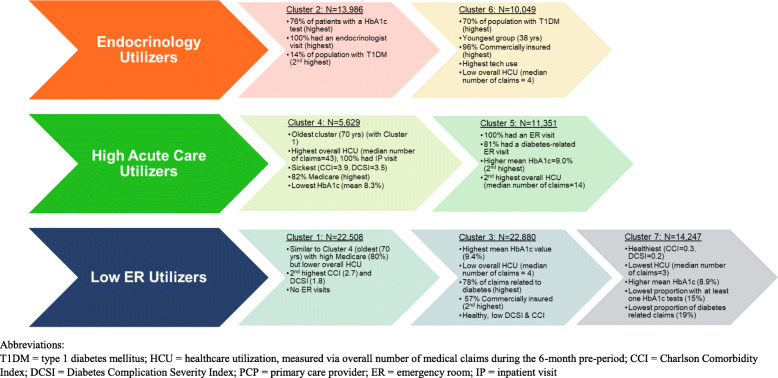
Cluster descriptions

The seven clusters fell into three main groupings in hierarchical order (Fig. 3**)**: those with endocrinology visits (Clusters 2 and 6) with differing concordant burden profiles; those with high acute care utilization (Clusters 4 and 5) with differences in inpatient days and ER visits; and those with low ER utilization (Clusters 1, 3, and 7) with varying comorbidity burden, diabetes utilization, and complication profiles.

### Endocrinology utilizers clusters

Cluster 2 all had at least one endocrinologist visit (mean = 1.8 visits/patient) and the average number of concordant comorbidities was 4.6. Cluster 6 had the youngest mean age (38.3 years) and the second highest utilization of endocrinologists (mean = 0.6 visits/patient) but had few concordant comorbidities (mean = 0.5). Additionally, the DCSI and CCI scores were relatively low.

### High acute care utilizers clusters

Those in Cluster 4 had the highest number of inpatient days with nearly all (99.1%) visiting the hospital at least once with the vast majority having an inpatient stay that was diabetes related (95.7%). Cluster 4 also had the highest overall number of medical claims and the highest comorbidity and complications burden (per the CCI and DCSI). Cluster 5 all experienced an ER visit, usually related to diabetes (80.8%).

### Low ER utilizers clusters

Those people in Clusters 1, 3, and 7 had few ER and endocrinology visits. Cluster 1 had a relatively high complication and comorbidity burden, second only to DCSI and CCI scores in Cluster 4, but those people in Cluster 1 had no ER utilization. In contrast, Clusters 3 and 7 had relatively low CCI and DCSI scores. Cluster 7 had the lowest overall number of medical claims (median = 3.00) and the lowest CCI scores (mean = 0.30) suggesting that Cluster 7 was the healthiest population, however only 15.2% of those in Cluster 7 had at least one HbA1c test performed. While those in Cluster 7 had nearly no diabetes-related claims (mean = 0.19), more than three-quarters of the claims for Cluster 3 were diabetes related (mean = 0.78).

Other notable differences were observed across clusters for variables not included in the cluster formations. Clusters 6 and 2 had the highest proportion of T1DM patients (69.6 and 13.6%, respectively) and diabetes technology use (pump, CGM, or BGM). Consistent with the young mean age, nearly all patients in Cluster 6 had commercial insurance. The highest proportion of Medicare Advantage members (and oldest mean age) was in Clusters 1 and 4 (> 80%). The highest mean HbA1c value (among the subset of the population with available values) was in Cluster 3 (9.4%). On the other hand, the lowest mean HbA1c of 8.3% was observed in Cluster 4, which also had the highest proportion without prior insulin use in the pre-period and the lowest mean number of months where insulin was filled.

### Multivariable modeling analysis

**Fig. 4:**
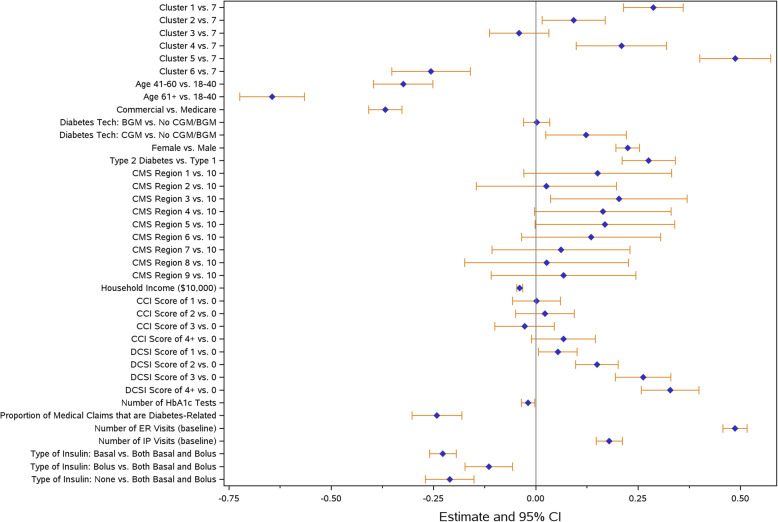
All-cause emergency room visits - model results. Abbreviations: BGM = blood glucose monitor; CCI = Charlson Comorbidity Index; CGM = continuous glucose monitor; DCSI = diabetes complications severity index; ER = emergency room; HbA1c = hemoglobin A1c; IP = inpatient

**Fig. 5:**
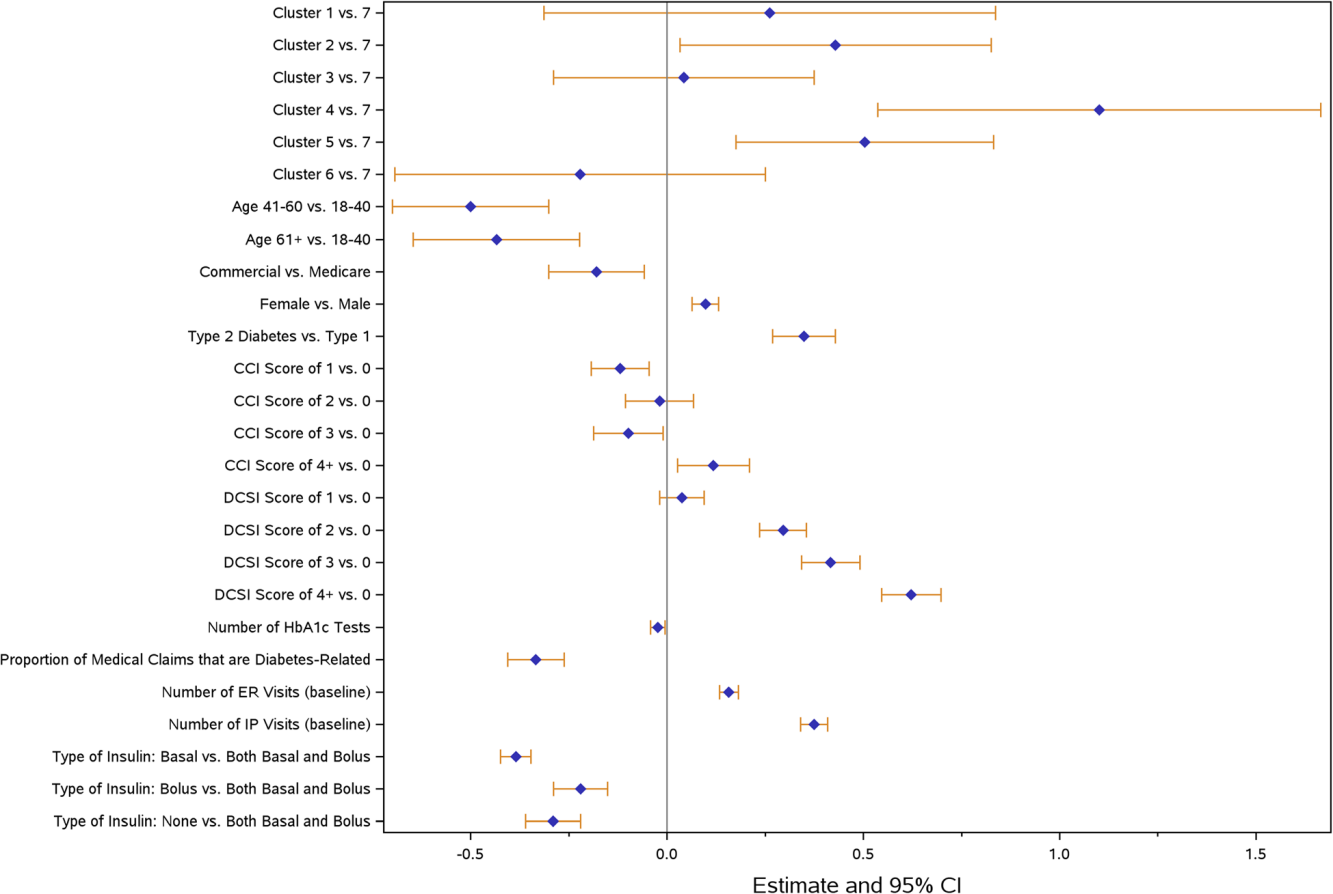
All-cause inpatient hospitalization - model results. Abbreviations: BGM = blood glucose monitor; CCI = Charlson Comorbidity Index; CGM = continuous glucose monitor; DCSI = diabetes complications severity index; ER = emergency room; HbA1c = hemoglobin A1c; IP = inpatient

**Fig. 6:**
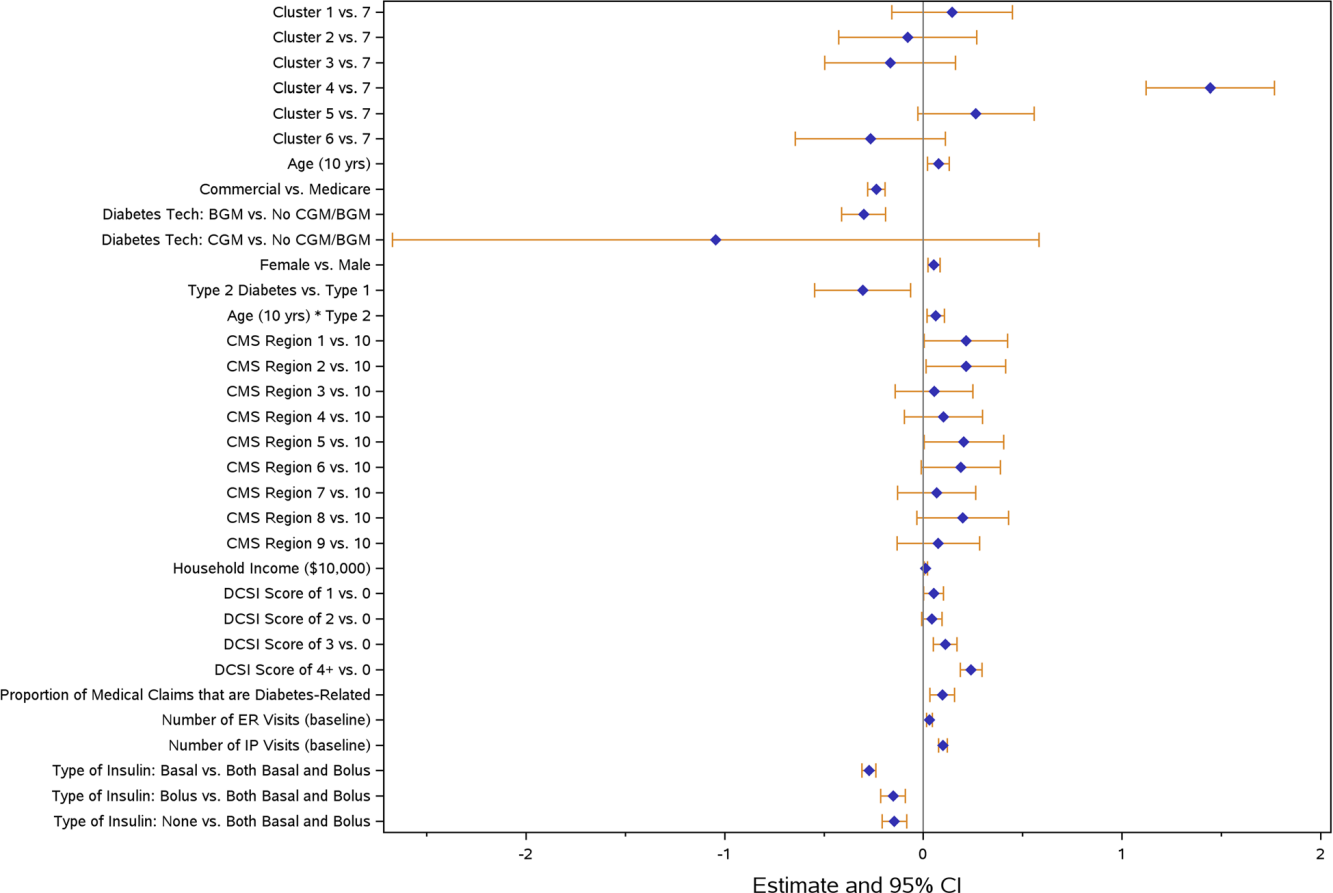
Total inpatient days - model results. Abbreviations: BGM = blood glucose monitor; CCI = Charlson Comorbidity Index; CGM = continuous glucose monitor; DCSI = diabetes complications severity index; ER = emergency room; HbA1c = hemoglobin A1c; IP = inpatient

For the multivariable modeling analysis, three key outcomes were chosen including all-cause ER visit (Fig. 4), all-cause inpatient hospitalization (Fig. 5), and total inpatient days (Fig. 6) during the follow-up period. Across all models, clusters were independently associated with the outcomes of interest after controlling for covariates that were potentially related to cluster assignment and/or outcomes of interest. Clusters 1, 2, 4, and 5 had higher odds of an ER visit, whereas Cluster 6 had lower odds compared to Cluster 7. Clusters 2, 4, and 5 also had higher odds of having a hospital admission. If hospitalized, Cluster 4 had significantly increased total inpatient days compared to Cluster 7.

## Discussion and conclusions

The aim of this study was to determine how clusters or subgroups of insulin-treated people with diabetes, based upon healthcare resource utilization, select social demographic/clinical characteristics, and diabetes management parameters, are related to health outcomes including acute care (ER and hospital inpatient) visits and total inpatient days. We did this study to help identify groups of patients that may be amenable to emerging diabetes management technologies. In this study, we identified seven clusters of insulin-treated people with diabetes, which have different patterns of healthcare utilization and diagnosed comorbidities in a large healthcare claims database. The most important factors in defining the clusters were the number of endocrinology visits, total inpatient days, concordant comorbidities, number of ER visits, comorbidity burden as measured by CCI and DCSI scores, and percentage of diabetes-related medical claims. Multivariable modeling showed that these clusters are significantly associated with ER visits, inpatient hospitalizations, and total inpatient days, suggesting that this approach may help identify patients at greater need for targeted disease management efforts at the population level. The clusters also offer providers clinically relevant information regarding treatment decisions for a patient population with diabetes.

Cluster analyses can reveal how variables, in our case administrative claims from people with diabetes, are related in complex datasets. The use of cluster analyses in healthcare decision making is still relatively uncommon but appears to be gaining acceptance [18–23]. Our work builds upon a few previously published cluster analyses in diabetes, which focused on readiness of CGM and other diabetes-related devices, self-management patterns in a pediatric population, factors influencing people with diabetes who have poorly controlled conditions, and identifying diabetes phenotypes [23–27]. These previous studies involved smaller numbers of participants from relatively homogeneous populations (e.g., T1DM registry) and/or more controlled conditions (e.g., clinical trial). In contrast, our study used a large healthcare claims database to evaluate whether routinely available data could identify relevant subgroups of insulin users.

Not only did clusters differ with respect to the specific variables used to form them (by design), but also on important other characteristics. Clusters 2 and 6 were formed primarily based on the use of endocrinologists. Not surprisingly, these were the clusters with the highest proportion of people with T1DM as well as utilizers of diabetes technology (pump, BGM, or CGM). Those in Cluster 2, however, had higher comorbidity burden and mean number of HbA1c tests than those in Cluster 6, but there was little difference in mean HbA1c values for these two clusters.

Two clusters were identified with high levels of acute care utilization. Those in Cluster 4 had the highest total inpatient days and everyone in Cluster 5 had an ER visit. These clusters differed, however, in their comorbidity burdens and glycemic control. Interestingly, the lowest mean observed HbA1c value was for Cluster 4, with the highest levels of overall medical utilization (median of 43.0 claims), acute care utilization (99.1% had an inpatient hospitalization), and highest CCI and DCSI scores. These results could suggest that a high burden of comorbidities or diabetes complications and increased interactions with hospitals facilitated more intensive diabetes management. However, because HbA1c values were only available on a subset of the study population (approximately 30%), additional analyses on datasets with more complete HbA1c data are needed to confirm this finding.

Conversely, higher mean HbA1c’s were observed among Clusters 3, 5, and 7 (in order of highest to lowest values). Clusters 3 and 7 differ from Cluster 5 in that they fell into the low utilization grouping (both acute care and overall utilization via number of medical claims) and had among the lowest CCI and DCSI scores. They differed from each other in one key aspect: the proportion of medical claims that were diabetes-related. Approximately three-fourths of the claims for Cluster 3 were related to diabetes, compared to less than 20% in Cluster 7. Because the CCI and DCSI scores are derived from the presence of diagnosis codes in claims data, on one hand it is not surprising for these clusters who have the lowest overall number of medical claims to have the lowest scores due to fewer opportunities to derive those diagnoses. But, on the other hand, the lack of diagnoses of comorbidities in the observed claims or lack of encounters altogether could suggest a healthier underlying population. Either way, the relatively high observed HbA1c values along with the low rates of interactions with healthcare providers suggested suboptimal diabetes self-management.

The current study demonstrated that even after adjusting for other covariates, cluster assignment was significantly predictive of future outcomes. Specifically, cluster assignment was associated with the likelihood of experiencing an ER or hospital inpatient visit and the total number of inpatient days for those with an admission. These results suggest that the specific combination of variables used in the cluster formations shed additional light onto the risk of untoward outcomes above and beyond traditional risk stratification, for example, based upon parameters including diabetes type, age, and HbA1c.

Furthermore, as these clusters were derived from variables routinely found in healthcare claims data where detailed clinical data are often missing, this approach could aid healthcare payers with population management efforts. We found some clusters utilizing less healthcare resources had higher observed mean HbA1c levels. This finding could suggest population management efforts in diabetes that are targeted at some of the lower healthcare utilizers in efforts to improve glycemic control, which could yield better long-term health outcomes for patients and improved quality metric ratings for providers and payers.

This study has limitations that should be considered. The cluster analysis was based on administrative claims data that rely on diagnostic codes entered by the healthcare provider for billing purposes. As such, they are proxies for clinical outcomes and may be prone to data coding errors or inaccuracies in patient records. Within this particular population, patients may have diagnostic codes for both type 1 and type 2 diabetes during the course of the study period, and while our algorithm to classify patients considered the preponderance of diagnostic codes among all available, in conjunction with treatment patterns common to each type of diabetes, it did not preclude the possibility of misclassification. Additionally, as of October 2015, all claims switched from ICD-9 to ICD-10 (study period January 2015 to June 2018) [28]. This switch possibly could have led to inaccuracies in coding due to unfamiliarity with the new system or mistakes in cross walking codes from ICD-9 to ICD-10 by providers. This potential for error should have had limited impact since condition identification was based on families of codes with multiple codes within a family and not on any single code. The comparisons of HbA1c values were incomplete as only a subset of patients (~ 30%) had values in the database. A number of relevant risk factors, including insulin dosing, diet, and exercise, were not available in the database.

Despite the limitations, this study was based on a well-developed study design and included data from a large number of insulin-using people with either T1DM or T2DM. Evaluating the impact of patient-reported outcomes and more socioeconomic data on cluster formations would be of interest to study in the future.

## Conclusion

In conclusion, we demonstrated that clustering analyses of healthcare claims data identified meaningful subgroups of patients that differed in comorbidity burden, healthcare utilization, and demographic factors. These clusters were found to be significantly associated with future outcomes indicating that providers and population health managers may be able to better estimate risk, based upon combinations of specific variables, and modify and/or personalize treatment accordingly.

## Supplementary Information

**Additional file 1 Table S1.** Classification of top 20 baseline comorbidities.

## Data Availability

The datasets generated and/or analyzed during the current study are not publicly available due to their proprietary nature, having been licensed only for the current study, and their protection under the Health Insurance Portability and Accountability Act (HIPAA). Subject to the data owner’s determination of reasonableness and only after execution of a confidentiality agreement with the data owner, among other requirements, the data is available from the corresponding author upon reasonable request.

## Abbreviations

BGM: blood glucose monitor
CART: classification and regression trees
CCI: Charlson comorbidity index
CGM: continuous glucose monitoring
DCSI: diabetes complications severity index
ER: emergency room
HbA1c: hemoglobin A1c
ICD: International Classification of Diseases
IP: inpatient
PCP: primary care physician
SD: standard deviation
T1DM: type 1 diabetes mellitus
T2DM: type 2 diabetes mellitus
US, USA: United States/United States of America
