# Supplementary material for: Evaluating the relationship between clinical and demographic characteristics of insulin-using people with diabetes and their health outcomes: a cluster analysis application
Source: BMC Health Serv Res. 2021 Jul 8;21:669. doi: 10.1186/s12913-021-06603-0 (PMC8265072; doi:10.1186/s12913-021-06603-0)
Supplement: Supplementary file 1 — Additional file 1 Table S1. Classification of top 20 baseline comorbidities. [file 12913_2021_6603_MOESM1_ESM.docx]

**Evaluating the relationship between clinical and demographic characteristics of insulin-using people with diabetes and their health outcomes: a cluster analysis application**

Elizabeth L Eby^1^, Alison Edwards^2^, Eric Meadows^1^, Ilya Lipkovich^1^, Brian D Benneyworth^1^, Kenneth Snow^2^

^1^ Eli Lilly and Company, Lilly Corporate Center, Indianapolis IN 46285, USA

^2^ Healthagen LLC (renamed CVS Health Clinical Trial Services LLC, effective 01 November 2020), 151 Farmington Avenue, Hartford CT 06156, USA

Corresponding author:

Elizabeth L Eby
Eli Lilly and Company
Lilly Corporate Center
Indianapolis, IN 46285, USA
Phone: 317.498.4328
Email: ebye@lilly.com

**Supplemental Materials 1**

**Table S1 Classification of top 20 baseline comorbidities**

| **T1DM (N = 11,826)** | **n (%)** | **Category** | **T2DM (N = 88,824)** | **n (%)** | **Category** |
| --- | --- | --- | --- | --- | --- |
| **Hyperlipidemia** | **6,824 (58%)** | **Discordant** | Hyperlipidemia | 79,743 (90%) | Concordant |
| **Hypertension** | **5,418 (46%)** | **Discordant** | Hypertension | 79,631 (90%) | Concordant |
| Chronic Thyroid Disorders | 3,566 (30%) | Concordant | Nonspecific Gastritis/Dyspepsia | 34,126 (38%) | Concordant |
| **Nonspecific Gastritis/Dyspepsia** | **2,103 (18%)** | **Discordant** | Obesity | 31,515 (35%) | Concordant |
| Depression | 1,850 (16%) | Concordant | Chronic Renal Failure | 26,482 (30%) | Concordant |
| **Low Back Pain** | **1,443 (12%)** | **Discordant** | Ischemic Heart Disease | 26,200 (29%) | Concordant |
| **Cataract** | **1,336 (11%)** | **Discordant** | Low Back Pain | 22,785 (26%) | Concordant |
| **Obesity** | **1,236 (10%)** | **Discordant** | Heart Failure | 21,811 (25%) | Concordant |
| **Anxiety** | **1,079 (9%)** | **Discordant** | **Chronic Thyroid Disorders** | **21,624 (24%)** | **Discordant** |
| Ischemic Heart Disease | 1,057 (9%) | Concordant | **Cataract** | **20,823 (23%)** | **Discordant** |
| Chronic Renal Failure | 1,015 (9%) | Concordant | Depression | 19,909 (22%) | Concordant |
| Glaucoma | 935 (8%) | Concordant | Osteoarthritis | 16,572 (19%) | Concordant |
| **Allergy** | **811 (7%)** | **Discordant** | Peripheral Artery Disease | 14,459 (16%) | Concordant |
| **Osteoarthritis** | **619 (5%)** | **Discordant** | **Chronic Obstructive Pulmonary Disease** | **13,770 (16%)** | **Discordant** |
| Heart Failure | 567 (5%) | Concordant | Cerebrovascular Disease | 13,482 (15%) | Concordant |
| Peripheral Artery Disease | 519 (4%) | Concordant | Atrial Fibrillation | 12,796 (14%) | Concordant |
| Cerebrovascular Disease | 478 (4%) | Concordant | Glaucoma | 12,601 (14%) | Concordant |
| **Benign Prostatic Hypertrophy** | **447 (4%)** | **Discordant** | **Benign Prostatic Hypertrophy** | **12,149 (14%)** | **Discordant** |
| **Chronic Obstructive Pulmonary Disease** | **373 (3%)** | **Discordant** | **Allergy** | **9,417 (11%)** | **Discordant** |
| Atrial Fibrillation | 323 (3%) | Concordant | **Anxiety** | **8,413 (9%)** | **Discordant** |

Abbreviations: n = number of patients with valid observations; N = total number of patients included in the analysis population; T1DM = type 1 diabetes mellitus; T2DM = type 2 diabetes mellitus.
